# Joint dynamics and efficient initialization techniques for potentials and currents in the P2D battery model

**DOI:** 10.1038/s41598-025-99733-y

**Published:** 2025-05-12

**Authors:** Keivan Haghverdi, Dmitri L. Danilov, Grietus Mulder, Luis D. Couto, Rüdiger-A. Eichel

**Affiliations:** 1 Institute of Physical Chemistry, RWTH Aachen University, Aachen, 52074 Germany; 2 Faculty of Mechanical Engineering, RWTH Aachen University, Aachen, 52062 Germany; 3 VITO, Boeretang 200, 2400 Mol, Belgium; 4 Institute of Energy Technologies, Fundamental Electrochemistry (IET-1), Forschungszentrum Jülich, Jülich, 52425 Germany; 5 Eindhoven University of Technology, 5600 MB Eindhoven, the Netherlands; 6 EnergyVille, Thor park 8310, Genk, Belgium

**Keywords:** Li-ion battery, P2D model, Pseudo-two-dimensional, Porous electrode, Model order reduction, Shooting method, Electrochemistry, Batteries

## Abstract

Solving the physics-based pseudo-two-dimensional (P2D) models involves using iterative methods, such as the Newton or the shooting method to solve a boundary condition problem. To use these iterative methods effectively, it is imperative to transform the boundary condition problem into an initial condition problem. This, in turn, necessitates initializing certain parameters, often done by providing guess values. The choice of these initial guess values can significantly impact convergence speed. This study proposes an analytically derived linear solution for initializing these conditions as an approximate guess. The proposed approach is not only computationally efficient, enhancing convergence speed and overall performance of the P2D model, but also straightforward to implement, making it a practical solution.

## Introduction

The global energy transition necessitates a pivotal shift towards innovative and renewable energy technologies, leading to an increased demand for storage capacity. This surge in capacity requirement has spurred a prominent trend toward adopting batteries, with lithium-ion batteries emerging as a significant player. These batteries find applications in stationary energy storage and electric vehicles, underscoring their versatility and importance in modern energy ecosystems^1^.

To maximize the utilization of lithium-ion batteries, advanced modeling techniques are essential. One such model, the pseudo-two-dimensional (P2D) battery model, also referred to as Doyle-Fuller-Newman (DFN), contains a system of partial differential equations (PDEs), and algebraic equations. This model partitions battery dynamics into two key dimensions. One spatial dimension along the length of the battery and another pseudo-dimension that characterize the radial dimension within the active particles of the electrodes. This framework offers a robust foundation for analyzing battery dynamics, facilitating precise evaluation, and optimization of lithium-ion battery performance^2–9^. However, solving the P2D model equations require significant computational resources and suffers from poor computation speed. In a prior study by the same authors, Haghverdi et al.^10^, a model order reduction technique was introduced to mitigate the occurrence of infinite values in the electrolyte current during model iteration. By eliminating these redundant iterations, they demonstrated significant improvements in the computational speed of the P2D model. Building upon this foundation, the current study endeavors to refine the method by introducing an analytically derived initial estimation for current and potential. This advancement aims to replace the preliminary loop for estimating the initial guess values of potentials in solids and electrolytes, employing a linear approximation of the Butler-Volmer equation. Transitioning from an iterative loop to a concise mathematical expression for initial value estimation is anticipated to yield significant benefits in terms of computational speed.

## Statement of the problem

The P2D model relies on iterative solvers such as the Newton or the shooting method to achieve the solution. For a comprehensive understanding of the governing equations of the P2D model, Table A1 is included in Appendix A, providing a detailed listing of the equations governing the P2D model. The shooting method addresses boundary value problems by transforming them into initial value problems through random guesses for missing initial variables, such as the solid and electrolyte potentials ($\varphi _1$ and $\varphi _2$). Initially, it conducts a forward run, often employing techniques like Euler or Runge-Kutta’s, and then iteratively fine-tunes these initial estimates to align the calculated ionic current in the electrolyte phase (denoted as $i_2$) with its specified boundary condition^11^. This iterative refinement typically employs the bisection or similar root-finding methods and continues until the desired accuracy in the parameter $i_2(L_p)$ is attained. Upon achieving the target boundary value for $i_2$, confidence is established in the accuracy of the guess values utilized for potentials $\varphi _1$ and $\varphi _2$ in reaching this solution.

**Fig. 1:**
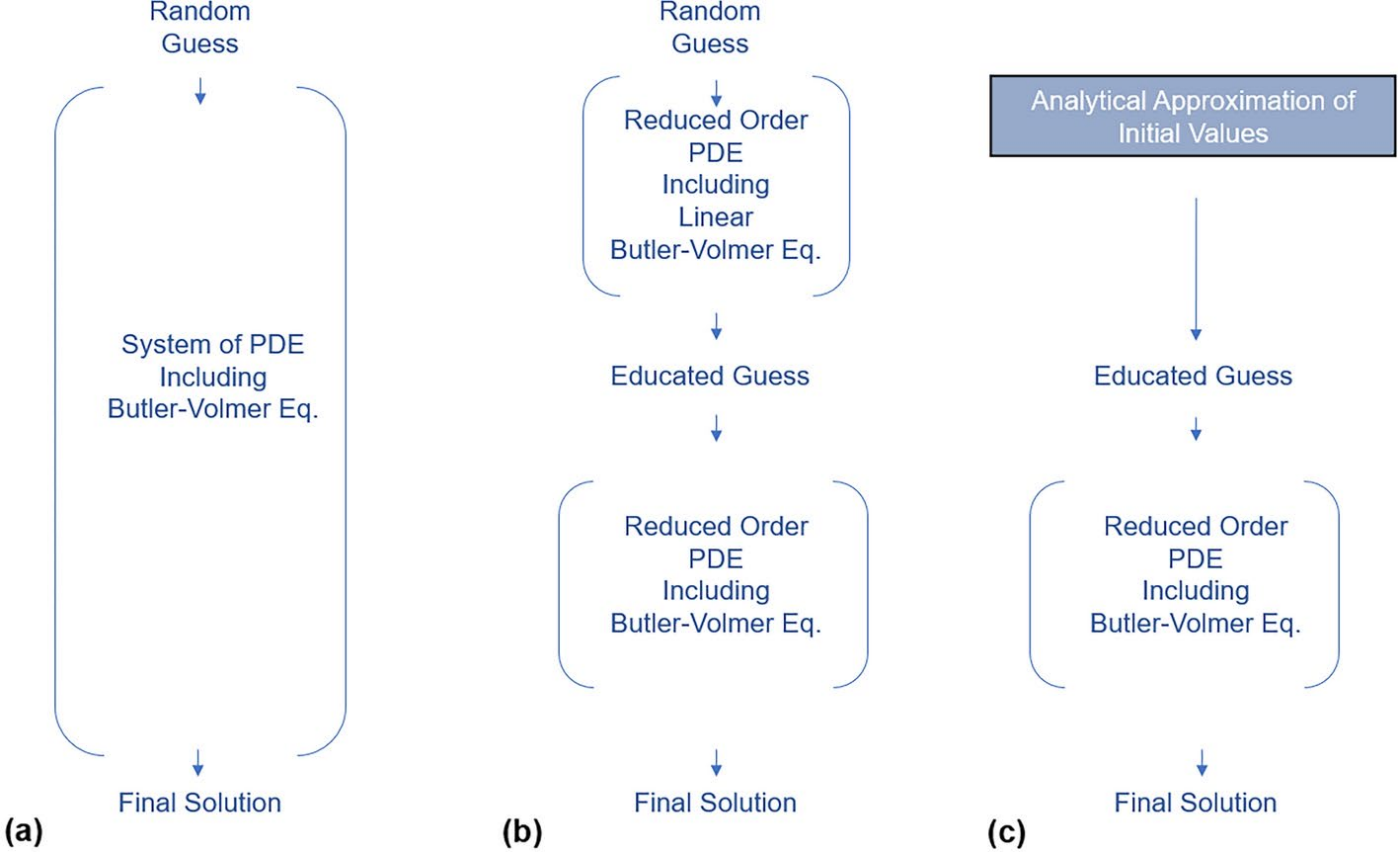
Solving the P2D model PDE strategies. (**a**) Original, (**b**) Preliminary linear Butler-Volmer loop for fast estimation of initial values^10^, (**c**) Replacing the preliminary loop with a direct analytical expression for faster calculation.

The effectiveness of iterative algorithms can be compromised when commencing simulations with arbitrary guesses for these potential values, resulting in diverging ionic currents within the electrolyte phase. This issue is addressed in the research conducted by Haghverdi et al.^10^. To tackle this challenge, a model order reduction technique was implemented to prevent the occurrence of infinite values and divergence during iterations. Additionally, the initial guess values were refined by employing a preliminary loop using linear Butler-Volmer approximation to estimate the initial value of $\psi = \varphi _1-\varphi _2$, which represents the galvanic pseudo-potential inside the cell. This study aims to replace the preliminary loop responsible for solving the system of PDEs with an analytical solution of the same PDE system. This substitution of the loop with an analytical expression is anticipated to accelerate the computation process for the P2D model. The evolution of strategies to solve this PDE system is illustrated in Fig. 1.

The battery model employed in this study is rooted in the research conducted by Chayambuka et al.^12,13^ and Chen et al.^14–16^. However, it is crucial to highlight that our research goes beyond the confines of these particular models, presenting a broadly applicable approach for all P2D battery models.

##  Model development

**Fig. 2:**
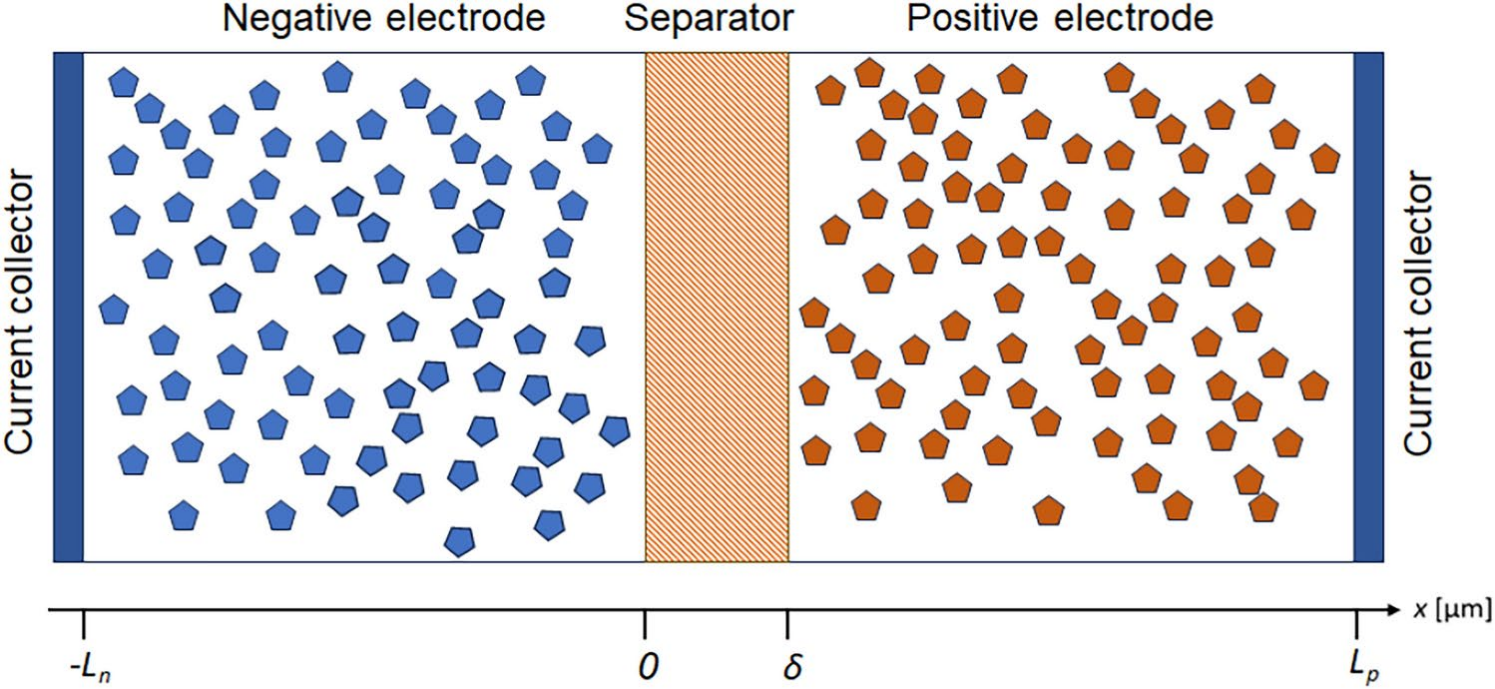
Schematic layout of the P2D model for a lithium-ion battery cell.

To simplify the mathematical presentation, this study focuses on the positive electrode equations. The equations for the negative electrode are identical, with the only difference being the adjustment of boundary conditions to reflect the negative electrode. Figure 2 provides a schematic representation of a battery cell, which includes current collectors on both sides, a negative electrode (typically graphite), a separator, and a positive electrode.

In this study, the origin of the *x*-axis is placed at the negative electrode/separator interface, with $x=0$, to simplify the mathematical presentation of the positive electrode for brevity. The solid hexagonal shapes represent active electrode particles. The parameter $\delta$ denotes the thickness of the separator membrane, while $L_p$ represents the position of the positive electrode current collector. The thickness of the positive porous electrode is given by $L_p - \delta$.

To numerically solve the P2D model, it is spatially and temporally discretized. Temporal discretization involves time-stepping, which transforms the system of partial differential equations into a system of ordinary differential equations (ODEs) at each time step. Subsequently, the ODEs need to be solved at each spatial point based on the chosen discretization method. For further details on P2D model solving strategies and discretization methods, refer to^17–21^. In this study, the spatial discretization of the model employs the finite difference method, further guided by a forward Euler method to traverse through each point.

Consider the cell at a certain moment during battery operation. Suppose that at that moment in time, current density *I* (A$\cdot \hbox {m}^{-2}$) is applied (index *t* is skipped for brevity). The question is how to determine the reaction rate distribution inside the porous electrode at the first moment when a current is applied. The system of equations for potentials and currents in both phases can then be written as:

1 $$\begin{aligned} i_1 = -\sigma _c \frac{d \varphi _1}{dx}, \end{aligned}$$

with boundary conditions

$$\begin{aligned} i_1(\delta )=0, \quad i_1(L_p)=I, \end{aligned}$$

and

2 $$\begin{aligned} i_2 = -\kappa _c\frac{d\varphi _2}{dx} + \frac{2\kappa _cRT}{F}(1-t_+)\frac{d \ln c_2}{d x}, \end{aligned}$$

with boundary conditions

$$\begin{aligned} i_2(\delta ) = I , \quad i_2(L_p)= 0. \end{aligned}$$

Here, $i_1$ and $\varphi _1$ represent the electronic current density (A$\cdot \hbox {m}^{-2}$) and electrical potential (V) within the porous electrode, while $i_2$ and $\varphi _2$ denote the ionic current density (A$\cdot \hbox {m}^{-2}$) and electrical potential (V) within the electrolyte residing inside the porous electrode. It is important to note that subscripts 1 and 2 correspond to the electrode (solid phase) and electrolyte (liquid phase), respectively. Furthermore, $\sigma _c$ and $\kappa _c$ stand for the effective electronic conductivity of the electrode and the effective ionic conductivity of the electrolyte (S$\cdot \hbox {m}^{-1}$), respectively. These effective conductivities consider the actual pathways through which species move within the porous media, with more detailed information available in the literature, particularly in the works of Doyle and Fuller et al.^8,9^.

The transfer of charge between the two phases is governed by Eq. (3), which is commonly referred to as the Butler-Volmer Equation.

3 $$\begin{aligned} \frac{di_2}{dx}= aFj_c = ai^0_c \left[ e^{\frac{\alpha F \eta ^{ct}_c}{RT}}-e^{-\frac{(1-\alpha )F \eta ^{ct}_c}{RT}} \right] \hspace{-1.2 mm}, \end{aligned}$$

where, *a* represents the specific area of the pores ($\hbox {m}^{-1}$), $j_c$ denotes the reaction rate (mol$\cdot \hbox {m}^{-2}\cdot \hbox {s}^{-1}$), and $i^0_c$ is the exchange current density (A$\cdot \hbox {m}^{-2}$). It is noteworthy that $j_c$ transforms into current density upon multiplication by the Faraday constant *F* (C$\cdot \hbox {mol}^{-1}$). The equation describing the charge transfer overpotential $\eta ^{ct}_c$ and its correlation with the potentials of the solid and electrolyte phases is presented in Eq. (4):

4 $$\begin{aligned} \eta ^{ct}_c= \varphi _1 - \varphi _2 - U_c(c^s_1, T), \end{aligned}$$

in this equation, $U_c$ represents the equilibrium potential of the electrode (V), while $c^s_1$ denotes the Li concentration at the surface of the electrode particle (mol$\cdot \hbox {m}^{-3}$) with spatial dependence. Additionally, *I* stands for the applied current density (A$\cdot \hbox {m}^{-2}$), and *R* and *T* represent the universal gas constant (J$\cdot \hbox {mol}^{-1}\cdot \hbox {K}^{-1}$) and absolute temperature (K), respectively. Finally, the conservation of charge is expressed by Eq. (5), as depicted below:

5 $$\begin{aligned} i_1+i_2=I. \end{aligned}$$

Suppose that the applied current density (*I*), and consequently the overpotential, is sufficiently small so that the Butler-Volmer equation Eq. (3) can be reduced to a linear relationship:

6 $$\begin{aligned} \frac{di_2}{dx}= \frac{F a \eta ^{ct}_c}{\rho } = \frac{F a}{\rho } (\varphi _1 - \varphi _2 - U_c(c^s_1,T)), \end{aligned}$$

where $\rho = \frac{RT}{i^0_c}$. By eliminating $i_1$ based on Eq. (5) and rearranging the equations, derived the following system of ordinary differential equations:

7 $$\begin{aligned} & \frac{d\varphi _1}{dx}=\frac{i_2 - I}{\sigma _c}, \end{aligned}$$

8 $$\begin{aligned} & \frac{d\varphi _2}{dx}= -\frac{i_2}{\kappa _c}+\frac{2RT}{F}(1-t_+)\frac{d \ln c_2}{d x}, \end{aligned}$$

9 $$\begin{aligned} & \frac{d i_2}{dx}= \frac{F a}{\rho }(\varphi _1 - \varphi _2 - U_c(c^s_1,T)). \end{aligned}$$

Next, by subtracting Eq. (8) from Eq. (7) and introducing the galvanic pseudo-potential $\psi = \varphi _1-\varphi _2$, the system of Eqs. (7)-(9) comes to the following form:

10 $$\begin{aligned} \frac{d \psi }{dx}= & \frac{i_2-I}{\sigma _c}+\frac{i_2}{\kappa _c}-\frac{2RT}{F}(1-t_+)\frac{d \ln c_2}{d x} \nonumber \\= & \frac{\kappa _c+\sigma _c}{\kappa _c \sigma _c}i_2-\frac{I}{\sigma _c}-\frac{2RT}{F}(1-t_+)\frac{d \ln c_2}{d x} \end{aligned}$$

11 $$\begin{aligned} \frac{di_2}{dx}= & \frac{F a}{\rho }(\psi -U_c(c^s_1,T)). \end{aligned}$$

Note that $U_c(c^s_1,T)=U_c(c^s_1(x),T)=U_c(x,T)$ is a function of coordinate *x* because the concentration in solid depends on *x*. Eqs. (10-11) can be rewritten in a matrix form. Denote state vector ${\varTheta }$, system matrix *A*, and right-hand side vector *b* as

12 $$\begin{aligned} \begin{aligned}&{\varTheta } = \begin{bmatrix} \psi \\ i_2 \end{bmatrix}&, \quad&A= \begin{bmatrix} 0 & \frac{\kappa _c+\sigma _c}{\kappa _c \sigma _c} \\ \\ \frac{F a}{\rho } & 0 \end{bmatrix}, \end{aligned} \end{aligned}$$

and

$$\begin{aligned} b=\begin{bmatrix} -\frac{I}{\sigma _c}-\frac{2RT}{F}(1-t_+)\frac{d ln c_2}{d x} \\ \\ -\frac{F a}{\rho }U_c(x) \end{bmatrix}, \end{aligned}$$

accordingly. Then Eqs. (10-11) can be written as

13 $$\begin{aligned} \frac{d {\varTheta }}{dx}= A {\varTheta } +b(x). \end{aligned}$$

As a first approximation the dependence of parameters $\kappa _c$ and $\sigma _c$ on *x* is ignored, they are treated as constants. The same assumption is made about $\rho$, at least when this parameter appears in matrix *A*. Eq. 13 is an inhomogeneous linear system of the first-order ODE-s. Note that vector *b* depends on *x*. To solve an inhomogeneous system of ODE-s one first must solve a homogeneous linear system, i.e. system

14 $$\begin{aligned} \frac{d {\varTheta }}{dx}= A {\varTheta }. \end{aligned}$$

Consider the characteristic equation

15 $$\begin{aligned} \det (A) = \begin{vmatrix} -\lambda&\frac{\kappa _c+\sigma _c}{\kappa _c \sigma _c} \\ \\ \frac{F a}{\rho }&-\lambda \end{vmatrix} = \lambda ^2- \frac{F a}{\rho }\left( \frac{1}{\sigma _c}+\frac{1}{\kappa _C}\right) =0. \end{aligned}$$

It has two simple roots, $\lambda _1= \sqrt{\frac{F a}{\rho }(\frac{1}{\sigma _c}+\frac{1}{\kappa _c})}$ and $\lambda _2= -\sqrt{\frac{F a}{\rho }(\frac{1}{\sigma _c}+\frac{1}{\kappa _c})}$. Denote $g=\sqrt{\frac{F a}{\rho }(\frac{1}{\sigma _c}+\frac{1}{\kappa _c})}$ to economize notations. The calculation of the eigenvector for $\lambda _1=g$ leads to a system of linear algebraic equations

16 $$\begin{aligned} \begin{bmatrix} -g & \frac{\kappa _c+\sigma _c}{\kappa _c \sigma _c} \\ \\ \frac{F a}{\rho } & -g \end{bmatrix} \begin{bmatrix} a_1 \\ a_2 \end{bmatrix} = \begin{bmatrix} -ga_1 + \frac{\kappa _c+\sigma _c}{\kappa _c \sigma _c} a_2 \\ \\ \frac{F a}{\rho }a_1 - g a_2 \end{bmatrix} = \begin{bmatrix} 0 \\ 0 \end{bmatrix}, \end{aligned}$$

which has a (non-normalized) solution $V_1=[\frac{\kappa _c+\sigma _c}{\kappa _c \sigma _c} \quad g]'$. Finally, the eigenvector $\lambda _2=-g$ brings $V_2=[-\frac{\kappa _c+\sigma _c}{\kappa _c \sigma _c} \quad g]'$ with similar calculations. Therefore, the general solution of Eq. (14) has a form

17 $$\begin{aligned} \widetilde{{\varTheta }}= \sum _{i=1}^{2} V_i e^{\lambda _1 x}= Z_1 e^{xg} \begin{bmatrix} \frac{\kappa _c+\sigma _c}{\kappa _c \sigma _c} \\ \\ g \end{bmatrix} + Z_2 e^{-xg} \begin{bmatrix} -\frac{\kappa _c+\sigma _c}{\kappa _c \sigma _c} \\ \\ g \end{bmatrix}, \end{aligned}$$

where $\widetilde{{\varTheta }}$ is a general solution to the homogenous Eq. (14), and $Z_i$ are constants. To obtain the solution of Eq. (13) these constants must be calculated. Consider the fundamental matrix of the solution, defined as

18 $$\begin{aligned} {\varOmega (x)}= & [ \omega _1(x) \quad \omega _2(x)] = [e^{xg}V_1 \quad e^{-xg} V_2] \nonumber \\= & \begin{bmatrix} \frac{\kappa _c+\sigma _c}{\kappa _c \sigma _c} e^{xg} & -\frac{\kappa _c+\sigma _c}{\kappa _c \sigma _c} e^{-xg} \\ \\ g e^{xg} & g e^{-xg} \end{bmatrix}. \end{aligned}$$

Then solution Eq. (17) can be written as $\widetilde{{\varTheta }}={\varOmega (x)}Z$, where $Z= (Z_1 \quad Z_2)'$. To extend the solution from a homogeneous system of equations to include non-homogeneous solutions, a vector *Z* that depends on the variable *x* must be considered. The solution of inhomogeneous Eq. (13) will take the form ${\varTheta }(x)={\varOmega }(x)Z(x)$. Substituting it into Eq. (13) leads to

19 $$\begin{aligned} \frac{d{\varTheta }}{dx}= \frac{d}{dx}{\varOmega }(x)Z(x)= \frac{d {\varOmega }(x)}{dx} Z(x)+ {\varOmega }(x)\frac{d Z(x)}{dx} \end{aligned}$$

$$\begin{aligned} = A {\varOmega }(x)Z(x)+{\varOmega }(x)\frac{dZ(x)}{dx}. \end{aligned}$$

This expression matches the right-hand side of Eq. (13), leading to the equation ${\varOmega }(x)\frac{dZ(x)}{dx}=b(x)$. Consequently,

20 $$\begin{aligned} \frac{dZ(x)}{dx}= {\varOmega }^{-1}(x)b(x), \end{aligned}$$

where

21 $$\begin{aligned} {\varOmega }^{-1}(x)&= \begin{bmatrix} \frac{\kappa _c+\sigma _c}{\kappa _c \sigma _c} e^{xg} & -\frac{\kappa _c+\sigma _c}{\kappa _c \sigma _c} e^{-xg} \\ \\ g e^{xg} & g e^{-xg} \end{bmatrix}^{-1} \nonumber \\&= \begin{bmatrix} \frac{\kappa _c\sigma _c}{\kappa _c+ \sigma _c} \frac{e^{-xg}}{2} & \frac{e^{-xg}}{2} \\ \\ \frac{\kappa _c \sigma _c}{\kappa _c+ \sigma _c} \frac{e^{xg}}{2} & \frac{e^{xg}}{2} \end{bmatrix}. \end{aligned}$$

In expanded form Eq. (20) can be written as

22 $$\begin{aligned} \frac{dZ(x)}{d}= & {\varOmega }^{-1}(x) \, b(x)\nonumber \\= & \begin{bmatrix} \frac{\kappa _c\sigma _c}{\kappa _c+ \sigma _c} \frac{e^{-xg}}{2} & \frac{e^{-xg}}{2} \\ \\ \frac{\kappa _c \sigma _c}{\kappa _c+ \sigma _c} \frac{e^{xg}}{2} & \frac{e^{xg}}{2} \end{bmatrix} \begin{bmatrix} -\frac{I}{\sigma _c}-\frac{2RT}{F}(1-t_+)\frac{d \ln c_2}{d x} \\ \\ -\frac{F a}{\rho }U_c(x) \end{bmatrix} \nonumber \\= & \begin{bmatrix} -\frac{I \kappa _c}{\kappa _c+\sigma _c}\frac{e^{-xg}}{2}-\frac{2RT}{F}(1-t_+)\frac{\kappa _c \sigma _c}{\kappa _c+\sigma _c}\frac{d \ln c_2}{d x}\frac{e^{-xg}}{2}-\frac{e^{-xg}}{2g}\frac{F a}{\rho } U_c(x) \\ \\ \frac{I \kappa _c}{\kappa _c+\sigma _c}\frac{e^{-xg}}{2}+\frac{2RT}{F}(1-t_+)\frac{\kappa _c \sigma _c}{\kappa _c+\sigma _c}\frac{d \ln c_2}{d x}\frac{e^{xg}}{2}-\frac{e^{xg}}{2g}\frac{F a}{\rho } U_c(x) \end{bmatrix}. \end{aligned}$$

To economize notation introduce functions representing all nonlinear terms in Eq. (22), according to the definitions

23 $$\begin{aligned} G_c(x)= \frac{2RT}{F}(1-t_+)\frac{\kappa _c \sigma _c}{\kappa _c+\sigma _c}\frac{d \ln c_2}{d x}, \end{aligned}$$

and

24 $$\begin{aligned} G_U(x)= \frac{F a}{g \rho } U_c(x), \end{aligned}$$

in these notations

25 $$\begin{aligned} \begin{bmatrix} \frac{dZ_1(x)}{dx} \\ \\ \frac{dZ_2(x)}{dx} \end{bmatrix} = \begin{bmatrix} -\frac{I \kappa _c}{\kappa _c+\sigma _c}\frac{e^{-xg}}{2} - \frac{e^{-xg}}{2}(G_c(x)+G_U(x)) \\ \\ \frac{I \kappa _c}{\kappa _c+\sigma _c}\frac{e^{xg}}{2} - \frac{e^{xg}}{2}(G_c(x)-G_U(x)) \end{bmatrix}, \end{aligned}$$

with an apparent solution

26 $$\begin{aligned} \begin{bmatrix} Z_1(x) \\ \\ Z_2(x) \end{bmatrix} = \begin{bmatrix} Z_1(\delta ) + \frac{I \kappa _c}{\kappa _c+\sigma _c}\frac{e^{-xg}-e^{-\delta g}}{2g} - \int \limits _{\delta }^{x}\frac{e^{-xg}}{2}(G_c(x)+G_U(x))dx \\ \\ Z_2(\delta ) + \frac{I \kappa _c}{\kappa _c+\sigma _c}\frac{e^{xg}-e^{\delta g}}{2g} + \int \limits _{\delta }^{x}\frac{e^{xg}}{2}(G_c(x)+G_U(x))dx \end{bmatrix}. \end{aligned}$$

It is important to note that the function $U_c(x)$, which represents the electrochemical potential alongside the *x*-axis, was inherently a function of concentration and temperature gradient alongside the *x*-axis $U_c(c^s_1,T)$. Given the exclusive focus on an approximation of the solution, to be used as the initial guess for the aforementioned equation-solving strategy shown in Fig. 1, the expressions can be simplified by assuming a uniform distribution of concentration along the *x*-axis. Consequently, this assumption leads to the reduction of $G_c$ to zero because $c_2$ is constant in the $\frac{d \ln c_2}{d x}$ expression, while $G_U$ becomes constant due to the constancy of $U_c(x)=U_c(c^s_1,T)$ in the absence of concentration gradient along *x*-axis. Therefore,

27 $$\begin{aligned} G_c(x) = 0, \end{aligned}$$

and

28 $$\begin{aligned} G_U(x) = G_U = \text {const}. \end{aligned}$$

The integral terms in the Eq. (31) become:

29 $$\begin{aligned} - \int \limits _{\delta }^{x}\frac{e^{-xg}}{2}(G_c(x)+G_U(x))dx= \frac{1}{2}{\textrm{e}}^{-g\,x} \,{\left( \delta -x\right) }G_\textit{U}, \end{aligned}$$

and

30 $$\begin{aligned} \int \limits _{\delta }^{x}\frac{e^{xg}}{2}(G_c(x)+G_U(x))dx= -\frac{1}{2}{\textrm{e}}^{-g\,x} \,{\left( \delta -x\right) G_\textit{U}}. \end{aligned}$$

Denote $z_1=Z_1(\delta )$ and $z_2=Z_2(\delta )$. That finally leads to a general solution of Eq. (13) in the form

31 $$\begin{aligned} {\varTheta }= & \begin{bmatrix} \psi (x) \\ \\ i_2(x) \end{bmatrix} = {\varOmega }(x)Z(x) \nonumber \\= & \begin{bmatrix} \frac{\kappa _c+\sigma _c}{\kappa _c \sigma _c} e^{xg} & -\frac{\kappa _c+\sigma _c}{\kappa _c \sigma _c} e^{-xg} \\ \\ g e^{xg} & g e^{-xg} \end{bmatrix} \begin{bmatrix} z_1 + \frac{I \kappa _c}{\kappa _c+\sigma _c}\frac{e^{-xg}-e^{-\delta g}}{2g} +\frac{1}{2}{\textrm{e}}^{-g\,x} \,{\left( \delta -x\right) G_\textit{U}} \\ \\ z_2 + \frac{I \kappa _c}{\kappa _c+\sigma _c}\frac{e^{xg}-e^{\delta g}}{2g} -\frac{1}{2}{\textrm{e}}^{-g\,x} \,{\left( \delta -x\right) G_\textit{U}} \end{bmatrix} \nonumber \\= & \left[ \begin{array}{c} \frac{{\textrm{e}}^{-g\,x} \,{\left( \kappa _c +\sigma _c \right) }\,\beta _2 }{\kappa _c \,\sigma _c }+\frac{{\textrm{e}}^{g\,x} \,{\left( \kappa _c +\sigma _c \right) }\,\beta _1 }{\kappa _c \,\sigma _c }\\ g\,{\textrm{e}}^{g\,x} \,\beta _1 -g\,{\textrm{e}}^{-g\,x} \,\beta _2 \end{array}\right] , \end{aligned}$$

where

32 $$\begin{aligned} \beta _1= & z_1 +\frac{G_U \,{\textrm{e}}^{-g\,x} \,{\left( \delta -x\right) }}{2}-\frac{I\,\kappa _c \,{\left( {\textrm{e}}^{-\delta \,g} -{\textrm{e}}^{-g\,x} \right) }}{2\,g\,{\left( \kappa _c +\sigma _c \right) }},\nonumber \\ \beta _2= & \frac{G_U \,{\textrm{e}}^{g\,x} \,{\left( \delta -x\right) }}{2}-z_2 +\frac{I\,\kappa _c \,{\left( {\textrm{e}}^{\delta \,g} -{\textrm{e}}^{g\,x} \right) }}{2\,g\,{\left( \kappa _c +\sigma _c \right) }}. \end{aligned}$$

Rewriting this result component-wise to obtain $\psi (x)$ and $i_2(x)$ yields

33 $$\begin{aligned} \begin{aligned} \psi (x)&= \frac{{\textrm{e}}^{-g\,x} \,{(\kappa _c +\sigma _c )}\,{\left( \frac{G_U \,{\textrm{e}}^{g\,x} \,{(\delta -x)}}{2}-z_2 +\frac{I\,\kappa _c \,{({\textrm{e}}^{\delta \,g} -{\textrm{e}}^{g\,x} )}}{\gamma _1 }\right) }}{\kappa _c \,\sigma _c }\\&\quad +\frac{{\textrm{e}}^{g\,x} \,{(\kappa _c +\sigma _c )}\,{\left( z_1 +\frac{G_U \,{\textrm{e}}^{-g\,x} \,{(\delta -x)}}{2}-\frac{I\,\kappa _c \,{({\textrm{e}}^{-\delta \,g} -{\textrm{e}}^{-g\,x} )}}{\gamma _1 }\right) }}{\kappa _c \,\sigma _c }, \end{aligned} \end{aligned}$$

and

34 $$\begin{aligned} \begin{aligned} i_2(x)&= g\,{\textrm{e}}^{g\,x} \,{\left( z_1 +\frac{G_U \,{\textrm{e}}^{-g\,x} \,{(\delta -x)}}{2}-\frac{I\,\kappa _c \,{({\textrm{e}}^{-\delta \,g} -{\textrm{e}}^{-g\,x} )}}{\gamma _1 }\right) }\\&\quad -g\,{\textrm{e}}^{-g\,x} \,{\left( \frac{G_U \,{\textrm{e}}^{g\,x} \,{(\delta -x)}}{2}-z_2 +\frac{I\,\kappa _c \,{({\textrm{e}}^{\delta \,g} -{\textrm{e}}^{g\,x} )}}{\gamma _1 }\right) }, \end{aligned} \end{aligned}$$

where $$\gamma _1 = 2\,g\,{(\kappa _c +\sigma _c )}$$

By utilizing the boundary conditions outlined in Eq. (2), where $i_2(\delta ) = I$ and $i_2(L_p)= 0$, the values of $z_1$ and $z_2$ can be calculated.

35 $$\begin{aligned} z_1= & -\frac{I\,\kappa _c \,{\textrm{e}}^{-L_p\,g} +2\,I\,\sigma _c \,{\textrm{e}}^{-L_p\,g} +2\,I\,\kappa _c \,{\textrm{e}}^{-\delta \,g} -I\,\kappa _c \,{\textrm{e}}^{L_p\,g} \,{\textrm{e}}^{-2\,\delta \,g} }{2\,g\,{\left( {\textrm{e}}^{L_p\,g} \,{\textrm{e}}^{-\delta \,g} -{\textrm{e}}^{-L_p\,g} \,{\textrm{e}}^{\delta \,g} \right) }\,{\left( \kappa _c +\sigma _c \right) }}, \nonumber \\ z_2= & \frac{I\,\kappa _c \,{\textrm{e}}^{L_p\,g} +2\,I\,\sigma _c \,{\textrm{e}}^{L_p\,g} +2\,I\,\kappa _c \,{\textrm{e}}^{\delta \,g} -I\,\kappa _c \,{\textrm{e}}^{-L_p\,g} \,{\textrm{e}}^{2\,\delta \,g} }{2\,g\,{\left( {\textrm{e}}^{L_p\,g} \,{\textrm{e}}^{-\delta \,g} -{\textrm{e}}^{-L_p\,g} \,{\textrm{e}}^{\delta \,g} \right) }\,{\left( \kappa _c +\sigma _c \right) }}. \end{aligned}$$

It is straightforward to calculate the units of $z_1$ and $z_2$ based on Eq. (35) which results in (A$\cdot \hbox {m}^{-2}$). Importing back the values of $z_1$ and $z_2$ in the Eq. (33) and Eq. (34) gives the final form of the $\psi (x)$ and $i_2(x)$

36 $$\begin{aligned} \psi (x) =&\frac{G_U \delta g}{g \kappa _c \sigma _c (e^{2L_pg} - e^{2\delta g})} \big ( \kappa _c e^{2L_pg} + \sigma _c e^{2L_pg} - g \kappa _c x e^{2L_pg} \nonumber \\&- \kappa _c e^{2\delta g} - g \sigma _c x e^{2L_pg} - \delta g \sigma _c e^{2\delta g} \nonumber \\&+ g \kappa _c x e^{2\delta g} + g \sigma _c x e^{2\delta g} \big ) \nonumber \\&- \frac{I}{g \kappa _c \sigma _c (e^{2L_pg} e^{gx} - e^{2\delta g} e^{gx})} \big ( \kappa _c e^{L_pg} e^{2\delta g} + \sigma _c e^{2L_pg} e^{\delta g} \nonumber \\&+ \kappa _c e^{L_pg} e^{2gx} + \sigma _c e^{\delta g} e^{2gx} \big ). \end{aligned}$$

37 $$\begin{aligned} i_2(x)=&\frac{I\,{\textrm{e}}^{-g\,x} \,{\left( \kappa _c \,{\textrm{e}}^{L_p\,g} +\sigma _c \,{\textrm{e}}^{\delta \,g} \right) }\,{\left( {\textrm{e}}^{2\,L_p\,g} -{\textrm{e}}^{2\,g\,x} \right) }}{{\left( \kappa _c +\sigma _c \right) }\,{\left( {\textrm{e}}^{2\,L_p\,g} -{\textrm{e}}^{2\,\delta \,g} \right) }}-\frac{I\,\kappa _c \,{\textrm{e}}^{-g\,x} \,{\left( {\textrm{e}}^{L_p\,g} -{\textrm{e}}^{g\,x} \right) }}{\kappa _c +\sigma _c }. \end{aligned}$$

The solutions provided in Eq. (36) and Eq. (37) are derived from the original system of ordinary differential equations. Utilizing these solutions, the values of $\psi (\delta )$ can serve as optimized initial guesses for the shooting method’s initial values.

## Results and discussion

The values utilized in the simulations of this study are detailed in Table 1, sourced from the research conducted by Chen et al.^22^.

**Table 1:** Model parameter values and units^22^.

Parameters	Values	Units	Description
*a*	$2.045 \cdot 10^5$	$\hbox {m}^{-1}$	Particles specific area
$i^0_c$	$6.328 \cdot 10^{-1}$	A$\cdot \hbox {m}^{-2}$	Exchange current density
*R*	8.314	J$\cdot \hbox {mol}^{-1}\cdot \hbox {K}^{-1}$	Universal gas constant
*T*	298	K	Temperature
*F*	$9.650 \cdot 10^4$	C$\cdot \hbox {mol}^{-1}$	Faraday constant
$\delta$	25	$\mu$m	Separator thickness
$L_p$	95	$\mu$m	Positive current collector position
*I*	$-9$	A$\cdot \hbox {m}^{-2}$	Applied current density
$\sigma _c$	$10^{-4} - 10^{-1}$	S$\cdot \hbox {m}^{-1}$	Electronic conductivity
$\kappa _c$	$10^{-4} - 10^{-1}$	S$\cdot \hbox {m}^{-1}$	Ionic conductivity
$t_{+}$	0.363	* -*	Transference number
$U_0(x,T)$	3.386	V	Electrode equilibrium potential

**Fig. 3:**
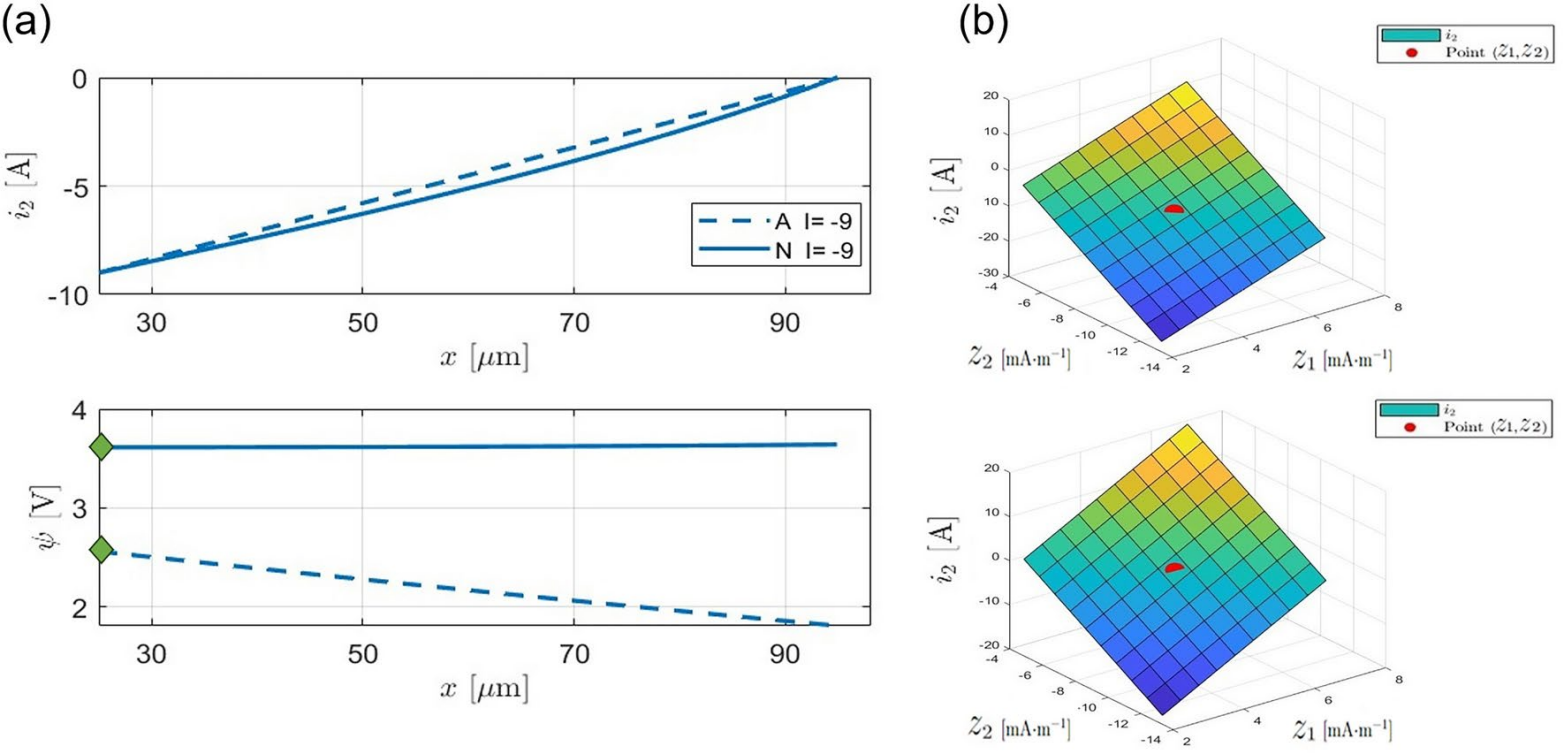
The evolution of $i_2$ (A$\cdot \hbox {m}^{-2}$) and $\psi$ (V) along *x* axis. (**a**) Top plot shows the analytical approximation of $i_2$ compared to numerically calculated $i_2$, Lower plot shows the analytical approximation of $\psi$ compared to numerically calculated $\psi$. Legend “A” and “N” stand for the analytical and the numerical solution. (**b**) $i_2$ values as a function of $z_1$ and $z_2$. The top plot is at boundary condition $x=\delta$, the lower plot is at boundary condition $x=L_p$. The red dot denotes the position of the analytically calculated $z_1$ and $z_2$ as defined in Eq. (35).

In Fig. 3 part (a), the evolution of $i_2$ and $\psi$ is depicted along the x-axis. The analytical solution for $i_2$ closely approximates the real values obtained numerically. In the lower plot of part (a) of Fig. 3, the focus is on identifying the initial point of the analytical $\psi$, which serves as a suitable starting point for the estimation process. This initial point aligns with the starting point of the numerical $\psi$, indicated by green diamonds.

In Fig. 3 part (b), the behavior of $i_2$ as a function of $z_1$ and $z_2$ is illustrated at positions $x = \delta$ and $x = L_p$, where the boundary conditions are defined by Eq. (2), respectively. It is apparent that except for the points computed in Eq. (35), alternative values for $z_1$ and $z_2$ fail to satisfy the boundary conditions.

**Fig. 4:**
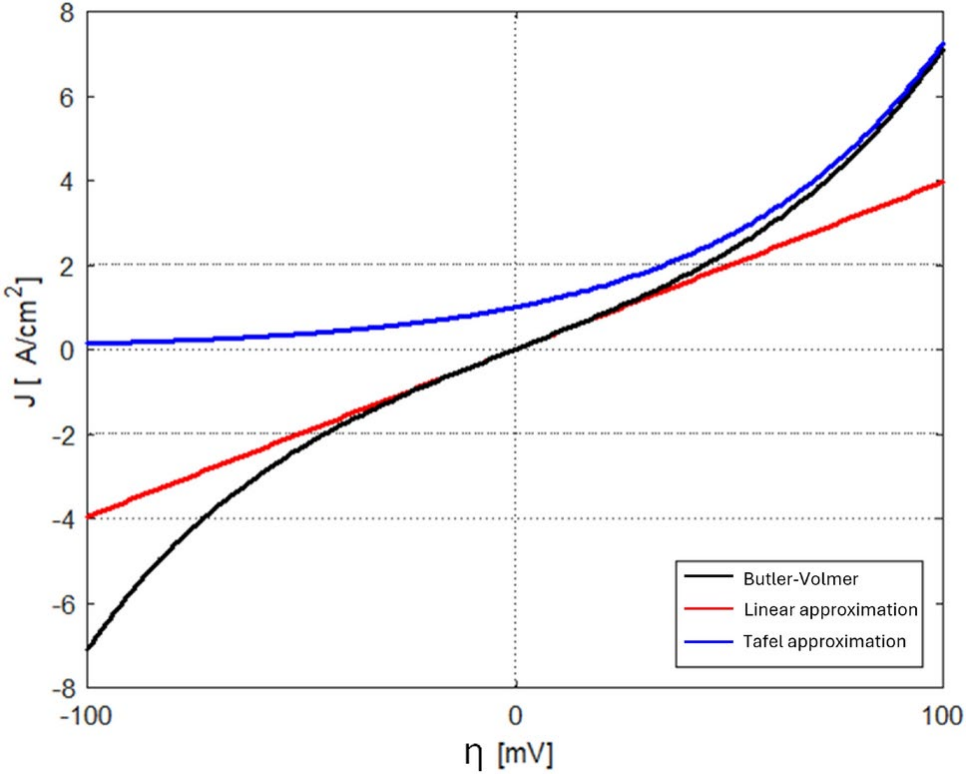
Comparison of the Butler-Volmer equation with its Tafel and linear approximations at $25\,^{\circ }$C.

In Fig. 4, the Butler-Volmer equation is compared with its Linear and Tafel approximations at $25\,^{\circ }$C. This low-current and low-overpotential region is identified based on this comparison, where the Butler-Volmer equation closely aligns with its linear form in an overpotential range of 100 mV. As illustrated in Fig. 1 part (c), the analytical expression derived in this study serves as an initial educated guess for the P2D system. When the operating conditions fall within or near the low-current and low-overpotential region, the improved model achieves the maximum speed improvement, as the analytical expression provides an initial guess very close to the actual solution, allowing the P2D model to converge instantly. However, as the operating conditions move further from this region, the accuracy of the analytical expression as an educated guess decreases. At extremely high currents, its effectiveness deteriorates to the point where it becomes no better than a random guess. Consequently, in such extreme conditions, the speed advantage of the method diminishes to zero, making it as slow as the traditional P2D model which uses random guess for initialization.

**Fig. 5:**
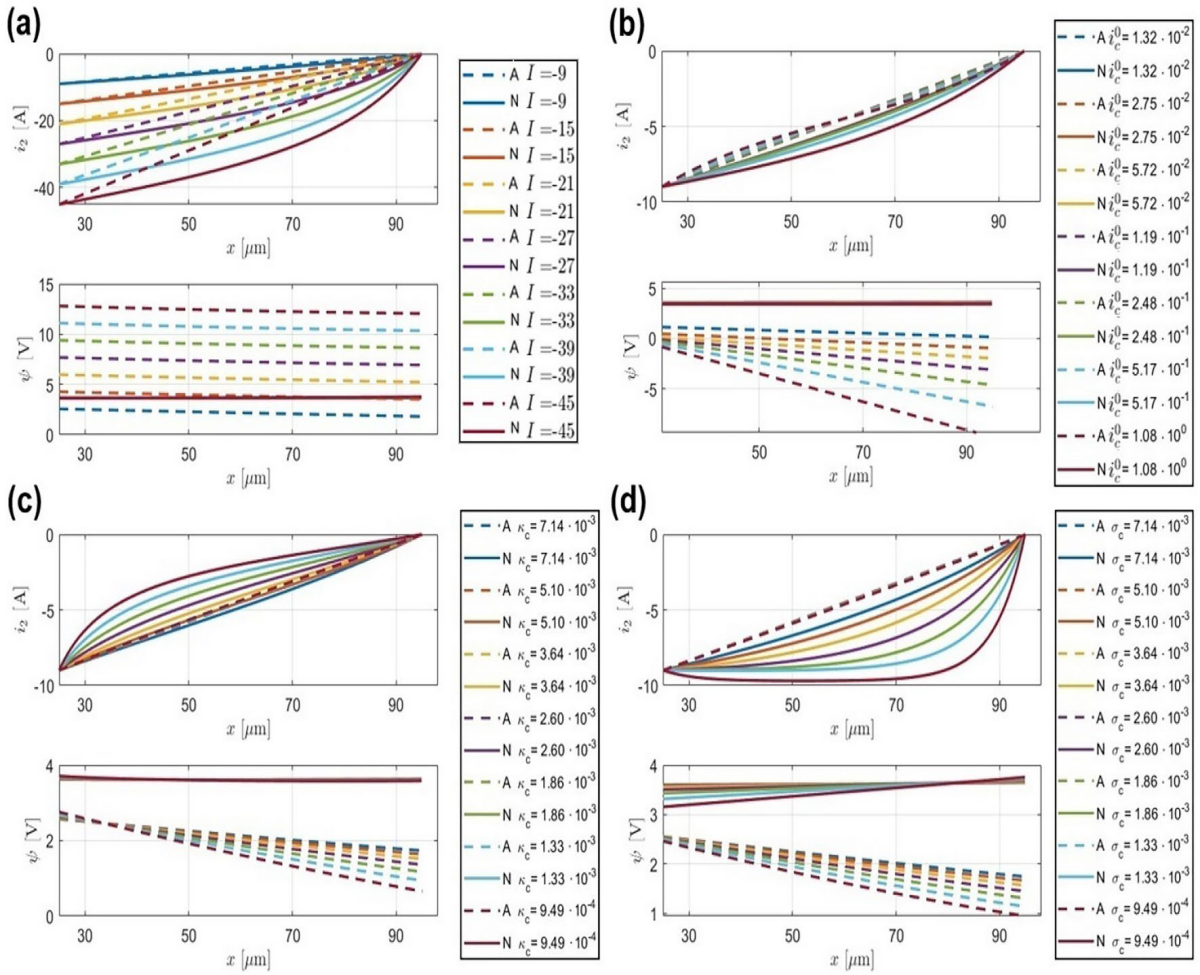
Sensitivity analysis of analytical and numerical $i_2$ and $\psi$ values with respect to various parameters. (**a**) $i_2$ and $\psi$ as a function of applied current density *I* (A$\cdot \hbox {m}^{-2}$). (**b**) $i_2$ and $\psi$ as a function of exchange current density $i^0_c$ (A$\cdot \hbox {m}^{-2}$). (**c**) $i_2$ and $\psi$ as a function of ionic conductivity $\kappa _c$ (S$\cdot \hbox {m}^{-1}$). (**d**) $i_2$ and $\psi$ as a function of electronic conductivity $\sigma _c$ (S$\cdot \hbox {m}^{-1}$). Legend “A” and “N” stand for the analytical and the numerical solution.

In Fig. 5, the behavior of $i_2$ and $\psi$ as a function of various parameters is depicted. In Fig. 5 part (a), the decrease in precision of the analytical solution as the applied current density increases is demonstrated. This outcome was anticipated, considering that the analytical solution was derived from the linear form of the Butler-Volmer equation, known for its higher accuracy in low current regions.

The sensitivity analysis with respect to $i^0_c$ is given in Fig. 5 part (b). The response of $i_2$ demonstrates commendable stability, showing only marginal error amplification with the increase of $i^0_c$. A corresponding trend is visible in the analytical solutions for $\psi$, where a diminishing accuracy is observed in comparison to their numerical counterparts as $i^0_c$ increases. Nevertheless, the analytical representation of $\psi$ remains firmly within an acceptable range, approximating the numerical solution. Therefore providing an approximation suitable to use as the initial value for the shooting method or the newtone method to enhance the computation speed for P2D models. More specifically, the values of $\psi$ at the boundary condition $x = 25 \mu m$ are used as the initial guess for the P2D model iterative solver. Hence, the precision of the estimated solution around this boundary region is more critical than in the rest of the $\psi$ function for this purpose.

In Fig. 5c, the sensitivity analysis regarding ionic conductivity $\kappa _c$ unveils a pattern similar to that observed with $i^0_c$. As $\kappa _c$ increases, there’s a reduction in the accuracy of the analytical solutions for $i_2$ and $\psi$, albeit they remain reliable approximations. This resilience qualifies them for incorporation into the proposed strategy as educated guesses for iterative solvers of the P2D model.

Similar logic extends to the sensitivity analysis of electronic conductivity $\sigma _c$, depicted in Fig. 5d. As $\sigma _c$ increases, a corresponding decrease in accuracy is observed in the behavior of functions $i_2$ and $\psi$. Despite this, the alignment between analytical and numerical representations of $i_2$ and $\psi$ remains robust across all plots, reinforcing their viability as dependable approximations even through parameter shifts.

Another question might be raised upon examining Fig. 5, given that the analytical solution for $i_2$ appears nearly linear in most cases and since the boundaries of $i_2$ are explicitly known as part of the boundary value problem, an alternative approach could involve approximating $i_2$ with a single straight line and calculating the $\psi$ values based on this simplified $i_2$ function afterward.

However, the issue with this idea lies in the fact that the governing equations of the system couple the potential $\psi (x)$ and the electrolyte current $i_2(x)$ intrinsically, meaning their evolution is interdependent across the entire domain. Specifically, these equations are of the form:

38 $$\begin{aligned} \frac{d\psi }{dx} = f(\psi , i_2), \quad \frac{di_2}{dx} = g(\psi , i_2), \end{aligned}$$

with the boundary conditions:

39 $$\begin{aligned} \psi (0) = \psi _0, \quad i_2(\delta ) = -I, \quad i_2(L_p) = 0. \end{aligned}$$

The shooting method is typically employed in this scenario, where it iteratively guesses the initial value $\psi (0)$, then solves the coupled equations for $\psi (x)$ and $i_2(x)$. In each iteration, the two variables $\psi (x)$ and $i_2(x)$ influence each other’s evolution, ensuring that they remain consistent across the entire domain. If the resulting $i_2(x)$ satisfies the boundary condition at $x = L_p$, the initial guess $\psi (0)$ is validated, and the solution is considered correct. This feedback mechanism between $\psi (x)$ and $i_2(x)$ guarantees that both quantities satisfy their respective governing equations and boundary conditions simultaneously.

**Fig. 6:**
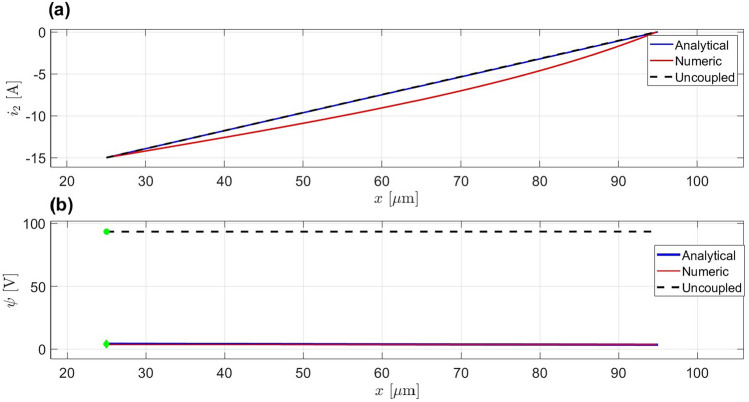
Comparison of numerical, analytical, and uncoupled linearized $i_2$ methods for $I = -15, [\text {A} \cdot \text {m}^{-2}]$. (**a**) The uncoupled linearized $i_2$ closely approximates both the analytical and numerical solutions. (**b**) The $\psi$ solutions for the analytical and numerical methods align well, while the uncoupled linearized method results in discrepancies, with random initiation points and increased error.

In contrast, when $i_2(x)$ is artificially imposed as a linear function:

$$\begin{aligned} i_2(x) = i_2(\delta ) + \left( \frac{i_2(L_p) - i_2(\delta )}{L_p} \right) x, \end{aligned}$$

the coupling between $\psi (x)$ and $i_2(x)$ is broken. In this approach, $i_2(x)$ is determined independently of $\psi (x)$, which means $\psi (x)$ is calculated based on a pre-determined $i_2(x)$, without adjusting $\psi (0)$ to satisfy the boundary condition at $x = \delta = 25$ $\mu$m. While the resulting shape of $\psi (x)$ may appear similar to the solution obtained via the coupled system, the absence of the feedback mechanism leads to a mismatch in the initial value of $\psi (0)$, which can make the solution unreliable with no way to determine the boundary of $\psi$ at $x = \delta = 25$ $\mu$m as shown in the Fig. 6.

When the fixed $i_2(x)$ is substituted into the governing equation for $\psi (x)$, the solution $\psi (x)$ is forced to conform to the imposed $i_2(x)$, which may result in a solution that satisfies the differential equation as* general solution* but fails to fully satisfy the original coupled system as *particular solution*.

The primary objective of this study is to obtain a reliable estimation of the initial potential $\psi (0)$ and current $i_2(x)$. However, the artificial imposition of an uncoupled linear $i_2(x)$ fails to achieve this goal, as it does not provide a robust mechanism for accurately determining the $\psi (0)$ initial value. This reinforces the importance of preserving the joint dynamics between $\psi (x)$ and $i_2(x)$ to ensure accurate and physically meaningful solutions.

It is important to emphasize that the strategy presented in this study also involves linearizing the problem (see Eq. 6). However, particular care is taken to preserve the coupled dynamics and interdependence between $\psi$ and $i_2$. By maintaining this relationship, the approach ensures a consistent and accurate representation of the joint behavior of these variables, leading to a reliable solution.

**Fig. 7:**
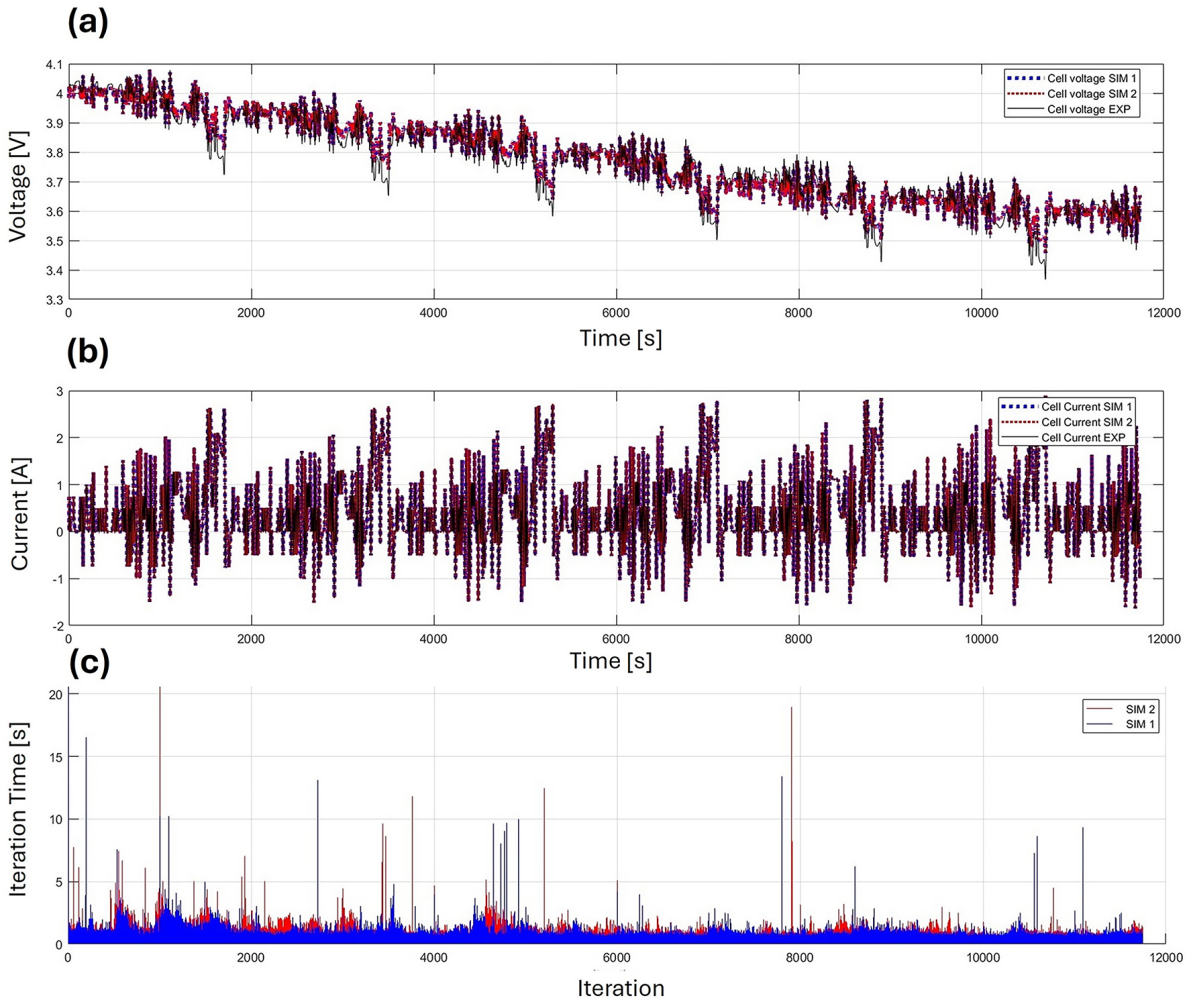
Comparison of accuracy and computational performance between the accelerated P2D (Sim 1) and base P2D (Sim 2) models for dynamic current. (**a**) Voltage output. (**b**) Current input. (**c**) Computation time per iteration.

Finally to demonstrate the proposed method performance, Fig. 7 illustrates the results of a model simulation under a dynamic current cycle lasting 12,000 seconds. The simulated battery has a capacity of 2.9 Ah, and the root mean square error (RMSE) of the voltage output was computed as 0.024 V. All simulations were conducted with a time step of one second. As expected, the cell voltage curves for both the base P2D model and the accelerated P2D model are identical, as both models solve the same set of equations. However, Fig. 7c highlights a significant reduction in iteration convergence time for the accelerated P2D model. On average, the original P2D simulation required 15.23 seconds to complete, while the accelerated version finished in just 11.87 seconds, resulting in a notable 22.06% improvement in computational efficiency.

In this dynamic current cycle, the C-rate did not exceed 1C, which contributed to the observed efficiency gain. It is important to note that in most industrial applications, manufacturers generally recommend charging rates lower than 1C.

**Fig. 8:**
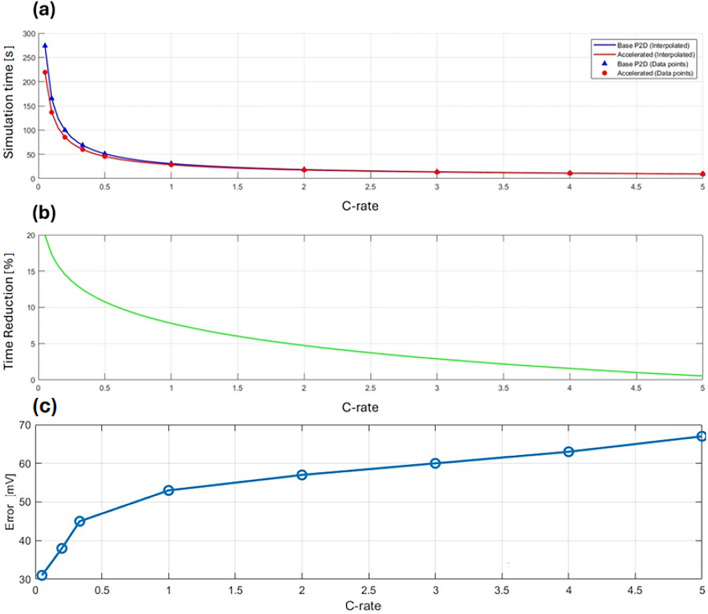
Comparison of computational performance between the accelerated P2D and base P2D models across different CC-CV cycles and C-rates. **(a)** Simulation time for each cycle at various C-rates. **(b)** Computation time reduction for each C-rate. **(c)** Computed voltage Root Mean Square Error (RMSE) for each C-rate.

To further evaluate performance, a computational time comparison was conducted for different constant current constant voltage (CCCV) cycles across various C-rates: C/20, C/10, C/5, C/3, C/2, 1C, 2C, 3C, 4C, and 5C. The simulation times for both the base and accelerated P2D models were recorded and plotted in Fig. 8a. Figure 8b shows the computational speedup gain in percentage for each C-rate. The points between the recorded data values were interpolated to estimate the acceleration gain for intermediate C-rates. Figure 8c illustrates the voltage error across different C-rates. These simulations were conducted on a Dell Latitude laptop with a Core i5 processor. It is clear that the accelerated P2D model outperforms the base P2D across all C-rate ranges, with a more significant improvement observed at lower C-rates compared to higher ones.

## Conclusion

This study introduces an analytical approach to enhance the optimization of initial values utilized in solving the P2D model equations via iterative solvers such as the shooting method or the Newton method. Analytically determining values for the current density in the electrolyte and the potentials in both the solid and electrolyte phases provides a reliable estimation of the initial values required to initiate an iterative solver of a P2D battery model. As this analytical expression replaces an ODE solving loop, it helps to accelerate the computational process necessary for P2D battery models.

Additionally, the obtained analytical solutions underwent a sensitivity analysis of the independent parameters, demonstrating their resilience and robustness. This analysis further supports the reliability of these approximations in solving both the P2D and accelerated P2D models.

The combined effect of the two strategies, as shown in Fig. 1, resulted in up to around 20% reduction in computation time at lower C-rates, with the benefit diminishing as the C-rate increases. Most industrial applications recommend charging regimes around 1C, where the model demonstrates an approximately 8% faster convergence time. As a complex system, advancements in improving the efficiency of the P2D model are often driven by incremental innovations, each addressing specific challenges or bottlenecks. These cumulative improvements are crucial in the ongoing effort to enhance computational efficiency.

A key advantage of the approach proposed in this study lies in its practicality and impact. Two direct analytical expressions for $\psi$ and $i_2$ through detailed analysis were derived, which can be seamlessly integrated into existing P2D models. Implementation is exceptionally straightforward, requiring only the replacement of a single line of code to substitute the random initialization of $\psi$ and $i_2$ with these analytical expressions. This approach strikes an excellent balance between minimal implementation effort and meaningful performance gains, making it a valuable contribution to the optimization of P2D models.

## Supplementary Information

Supplementary Information 1.

Supplementary Information 2.

Supplementary Information 3.

Supplementary Information 4.

## Data Availability

The datasets used during this study are available from the corresponding author upon reasonable request. Additionally, formulation details of the P2D model are provided in Appendix A.

## References

[1] Greim, P., Solomon, A. & Breyer, C. Assessment of lithium criticality in the global energy transition and addressing policy gaps in transportation. *Nat. Commun.* **11**(1), 4570 (2020). 32917866 10.1038/s41467-020-18402-y PMC7486911

[2] Newman, J. S. & Tobias, C. W. Theoretical analysis of current distribution in porous electrodes. *J. Electrochem. Soc.* **109**(12), 1183 (1962).

[3] Newman, J. & Tiedemann, W. Porous-electrode theory with battery applications. *AIChE J.* **21**(1), 25–41 (1975).

[4] Darling, R. & Newman, J. On the short-time behavior of porous intercalation electrodes. *J. Electrochem. Soc.* **144**(9), 3057 (1997).

[5] Scott, K. & Argyropoulos, P. A current distribution model of a porous fuel cell electrode. *J. Electroanal. Chem.* **567**(1), 103–109 (2004).

[6] Lanzi, O. & Landau, U. Effect of pore structure on current and potential distributions in a porous electrode. *J. Electrochem. Soc.* **137**(2), 585 (1990).

[7] Szpak, S. & Katan, T. An experimental study of reaction profiles in porous electrodes. *J. Electrochem. Soc.* **122**(8), 1063 (1975).

[8] Doyle, M., Fuller, T. F. & Newman, J. Modeling of galvanostatic charge and discharge of the lithium/polymer/insertion cell. *J. Electrochem. Soc.* **140**(6), 1526 (1993).

[9] Fuller, T. F., Doyle, M. & Newman, J. Simulation and optimization of the dual lithium ion insertion cell. *J. Electrochem. Soc.* **141**(1), 1 (1994).

[10] Haghverdi, K., Danilov, D. L., Mulder, G., Couto, L. D. & Eichel, R.-A. On the joint dynamics of potentials and currents in porous electrodes: Model reduction. *J. Power Sources Adv.* **26**, 100138 (2024).

[11] Osborne, M. R. On shooting methods for boundary value problems. *J. Math. Anal. Appl.* **27**(2), 417–433 (1969).

[12] Chayambuka, K., Mulder, G., Danilov, D. L. & Notten, P. H. Determination of state-of-charge dependent diffusion coefficients and kinetic rate constants of phase changing electrode materials using physics-based models. *J. Power Sources Adv.* **9**, 100056 (2021).

[13] Chayambuka, K., Mulder, G., Danilov, D. L. & Notten, P. H. A hybrid backward euler control volume method to solve the concentration dependent solid-state diffusion problem in battery modeling. *J. Appl. Math. Phys.* **8**(6), 1066–1080 (2020).

[14] Chen, Z. et al. Overpotential analysis of graphite-based li-ion batteries seen from a porous electrode modeling perspective. *J. Power Sources* **509**, 230345 (2021).

[15] Chen, Z., Danilov, D. L., Eichel, R.-A. & Notten, P. H. Li+ concentration waves in a liquid electrolyte of li-ion batteries with porous graphite-based electrodes. *Energy Storage Mater.* **48**, 475–486 (2022).

[16] Chen, Z., Danilov, D. L., Eichel, R.-A. & Notten, P. H. Porous electrode modeling and its applications to li-ion batteries. *Adv. Energy Mater.* **12**(32), 2201506 (2022).

[17] Hussain, A., Mao, Z., Li, M., Ahmed, M., Xu, W., Zhang, W. & Chen, Z. A comprehensive review of the pseudo-two-dimensional (p2d) model: Model development, solutions methods, and applications, *Advanced Theory and Simulations*, 2401016.

[18] Zeng, Y. et al. Efficient conservative numerical schemes for 1d nonlinear spherical diffusion equations with applications in battery modeling. *J. Electrochem. Soc.* **160**(9), A1565 (2013).

[19] Torchio, M., Magni, L., Gopaluni, R. B., Braatz, R. D. & Raimondo, D. M. Lionsimba: a matlab framework based on a finite volume model suitable for li-ion battery design, simulation, and control. *J. Electrochem. Soc.* **163**(7), A1192 (2016).

[20] Yin, X. & Zhang, D. batp2dfoam: An efficient segregated solver for the pseudo-2-dimensional (p2d) model of li-ion batteries. *J. Electrochem. Soc.* **170**(3), 030521 (2023).

[21] Oyewole, I., Kwak, K. H., Kim, Y. & Lin, X. Optimal discretization approach to the enhanced single-particle model for li-ion batteries. *IEEE Trans. Transp. Electrif.* **7**(2), 369–381 (2020).

[22] Chen, Z., Danilov, D. L., Eichel, R.-A. & Notten, P. H. On the reaction rate distribution in porous electrodes. *Electrochem. Commun.* **121**, 106865 (2020).

