# Supplementary material for: Joint dynamics and efficient initialization techniques for potentials and currents in the P2D battery model
Source: Sci Rep. 2025 May 12;15:16477. doi: 10.1038/s41598-025-99733-y (PMC12069546; doi:10.1038/s41598-025-99733-y)
Supplement: Supplementary file 1 — Supplementary Information 1. [file 41598_2025_99733_MOESM1_ESM.pdf]

Appendix A. Supplementary data

Table A1: P2D model equations

Name of Equation	Mathematical Equation
Diffusion in particles	$$\frac{\partial c_1}{\partial t} = \frac{1}{r^2}\frac{\partial}{\partial r}(r^2 D_1 \frac{\partial c_1}{\partial r})$$
Kinetics	$$i_c^0 = Fk(c_1^{max} - c_1^s)^{\alpha}(c_1^s)^{(1-\alpha)}(c_2)^{\alpha}$$ $$j = \frac{i_c^0}{F}\left[e^{\frac{\alpha F \eta^{ct}}{RT}} - e^{-\frac{(1-\alpha)F\eta^{ct}}{RT}}\right]$$
Mass balance	$$\epsilon\frac{\partial c_2}{\partial t} = \frac{\partial}{\partial x}\left(D_2^{eff}\frac{\partial c_2}{\partial x}\right) + aj(1-t_+)$$
Potential in solution	$$i_2 = -\kappa_c\frac{\partial \varphi_2}{\partial x} + \frac{2\kappa_c RT}{F}(1-t_+)\left(1 + \frac{dlnf_{\pm}}{dlnc_2}\right)\frac{\partial \ln c_2}{\partial x}$$
Potential in solid	$$i_1 = -\sigma_c\frac{\partial \varphi_1}{\partial x}$$ $$i_1 + i_2 = I$$ $$Faj = \frac{\partial i_2}{\partial x}$$
Battery output voltage	$$V_{bat} = \varphi_1\|_{x=L} - \varphi_1\|_{x=0} - IR_f$$
